# Chloroplast Genome Evolution in Pleurothallidinae (Orchidaceae): Lineage-Specific Selection, Codon Usage Patterns, and Phylogenetic Implications

**DOI:** 10.3390/genes17020199

**Published:** 2026-02-07

**Authors:** Yuxue Liu, Qiang Zhang, Zhenhua Wu, Zhenping Shi, Shuo Wang

**Affiliations:** 1 College of Biological Engineering, Qingdao University of Science and Technology, Qingdao 266042, China; 17860282396@139.com (Y.L.); zhangqiang@qust.edu.cn (Q.Z.); 18266568228@163.com (Z.W.); 2 College of Chemical Engineering, Qingdao University of Science and Technology, Qingdao 266042, China

**Keywords:** chloroplast genome, codon usage bias, molecular markers, Orchidaceae, phylogenomics, Pleurothallidinae, positive selection

## Abstract

Background: The subtribe Pleurothallidinae is a diverse group within Orchidaceae with a complex taxonomic history. Comparative plastome analysis can provide insights into genome evolution and facilitate phylogenetic reconstruction. Methods: Here we analyzed 25 complete chloroplast genomes representing 15 genera, including 14 newly assembled genomes, to investigate plastome evolution in this subtribe. Results: All genomes exhibited the typical quadripartite structure (148, 246–158, 138 bp) with conserved gene content (128–134 genes). While most protein-coding genes were under purifying selection, we detected signatures of positive selection in specific lineages. Notably, *ndhF* in *Lepanthes tachirensis* showed a markedly elevated Ka/Ks ratio (3.65), which may be associated with adaptation to an extensive distributional range. ENC-plot analysis indicated that natural selection, rather than mutation pressure alone, shapes codon usage bias, with patterns varying among species from different geographic regions. Nucleotide diversity analysis identified eight hypervariable intergenic regions (*psbK*-*psbI*, *atpI*-*rps2*, *petN*-*psbM*, *psbB*-*psbT*, *petD*-*rpoA*, *rpoA*-*rps11*, *rps3*-*rpl22*, *ccsA*-*ndhD*) suitable as candidate molecular markers. Phylogenetic analysis confirmed that *Lepanthes* and *Pleurothallis* are non-monophyletic as traditionally defined. Conclusions: These findings expand plastome resources for Pleurothallidinae, reveal lineage-specific patterns of selection, and provide molecular markers for future taxonomic and evolutionary studies.

## 1. Introduction

The subtribe Pleurothallidinae (Orchidaceae: Epidendroideae) comprises primarily epiphytic orchids distributed in Neotropical montane forests [1,2]. This subtribe has a complex taxonomic history, with generic boundaries repeatedly revised as molecular phylogenetic data have accumulated [2]. Many traditionally recognized genera have proven to be polyphyletic or paraphyletic, prompting ongoing efforts to establish classifications that reflect evolutionary relationships [1,3]. Molecular phylogenetic studies based on nuclear ITS and plastid *matK* sequences have confirmed the monophyly of Pleurothallidinae while revealing that several genera, including *Lepanthes* and *Pleurothallis*, are not monophyletic as traditionally circumscribed [1,2]. These findings highlight the need for additional molecular data to resolve phylogenetic relationships and facilitate taxonomic revision.

Complete chloroplast genome (plastome) sequences provide rich information for phylogenetic reconstruction and molecular evolutionary analysis [4,5,6]. Beyond phylogenetic signal, plastomes can reveal patterns of structural evolution, selection pressure on protein-coding genes, and codon usage bias that may reflect adaptation to environmental conditions [7]. The typical angiosperm plastome comprises a large single-copy (LSC) region, a small single-copy (SSC) region, and two inverted repeat (IR) regions [8,9]. Comparative plastome analysis has proven valuable for identifying hypervariable regions useful as molecular markers and for detecting signatures of adaptive evolution [10,11].

Although plastome sequences from several Pleurothallidinae genera have been reported [12], comprehensive comparative analyses examining selection pressures and codon usage patterns across multiple genera remain limited. In particular, whether plastome evolution in Pleurothallidinae shows lineage-specific patterns potentially associated with ecological adaptation has not been systematically investigated.

In this study, we assembled 14 new plastomes and analyzed them together with 11 previously published genomes, representing 15 genera and 25 species of Pleurothallidinae. Our objectives were to: (1) characterize plastome structural features and identify hypervariable regions suitable as molecular markers; (2) evaluate selective pressures on protein-coding genes and identify genes potentially under positive selection; (3) analyze codon usage patterns and assess potential factors shaping codon bias; and (4) reconstruct phylogenetic relationships and evaluate congruence with traditional classifications. This study aims to expand genomic resources for Pleurothallidinae and provide insights into plastome evolution in this taxonomically challenging subtribe.

## 2. Materials and Methods

### 2.1. Chloroplast Genome Assembly and Annotation

Raw sequencing reads for 30 Pleurothallidinae accessions were retrieved from the NCBI Sequence Read Archive (https://www.ncbi.nlm.nih.gov/). De novo chloroplast genome assembly was performed using GetOrganelle v1.7.7.0 [13] with default parameters. Fourteen complete plastomes were successfully assembled and annotated using GeSeq v1.42 [14]. Annotations were manually curated in Geneious v9.0.2 [15], with particular attention to intron boundaries and start/stop codon positions. Annotated genomes were formatted using GB2Sequin v16 [16] and deposited in GenBank (accession numbers: OR713737–OR713740, OR909686–OR909688, PP094516, PX828354–PX828359). Circular genome maps were generated using OGDRAW v1.3.1 [17].

### 2.2. Genome Structural Analysis

Plastome characteristics including region lengths, GC content, and gene composition were calculated using Geneious v9.0.2 [15,18]. Simple sequence repeats (SSRs) were identified using MISA [19] with minimum repeat thresholds of 9, 4, 3, 3, 3, and 3 for mono- through hexanucleotide motifs, respectively [20]. Tandem repeats were detected using Tandem Repeats Finder [21]. IR boundary positions were visualized using IRscope [22] with *Cephalanthera damasonium* (MH590345) as reference. Whole-genome alignments were performed using mVISTA [23] in Shuffle-LAGAN mode with *Anathallis microphyta* as reference.

### 2.3. Selection Pressure and Codon Usage Analysis

The ratio of non-synonymous to synonymous substitutions (Ka/Ks) was calculated for all shared protein-coding genes using *C. damasonium* as reference. Genes with Ka/Ks > 1 were identified as candidates potentially under positive selection. Relative synonymous codon usage (RSCU) values and effective number of codons (ENCs) were calculated using MEGA v11 [24]. Effective number of codons–GC content at the third codon position (ENC-GC3) plots were constructed following [25] to assess the relative contributions of mutation pressure and selection to codon usage bias. RSCU heatmaps were generated using TBtools v1.1047 [26].

### 2.4. Nucleotide Diversity and Marker Identification

Nucleotide diversity (π) was calculated using DnaSP v6.12 [27] with a sliding window approach (window length = 600 bp; step size = 50 bp). Regions with π > 0.025 for coding sequences or π > 0.05 for intergenic spacers were designated as hypervariable regions.

### 2.5. Phylogenetic Analysis

Phylogenetic relationships were inferred using plastome sequences from 57 species of tribe Epidendreae (Table S3), including representatives of subtribes Agrostophyllinae, Bletiinae, Calypsoinae, Laeliinae, and Pleurothallidinae. Based on previous phylogenetic studies, *C. damasonium* (MH590345) and *Cephalanthera rubra* (NC_041181) were selected as outgroups, as they belong to a clade phylogenetically distinct from Epidendreae within Epidendroideae, ensuring an appropriate evolutionary distance for rooting the phylogenetic tree [28]. For phylogenetic reconstruction, protein-coding sequences were extracted following strict criteria: (1) non-pseudogenized protein-coding genes shared across all 57 species were included to ensure data consistency; (2) duplicate gene copies from the two inverted repeat (IR) regions (IRA and IRB) were excluded. They were aligned using PhyloSuite [29,30] and trimmed using trimAl [31]. Maximum likelihood analysis was conducted in IQ-TREE v2.1.3 [32] with the GTR + I + G model selected by BIC. Branch support was assessed using 1000 ultrafast bootstrap replicates.

## 3. Results

### 3.1. General Features of Pleurothallidinae Chloroplast Genomes

**Figure 1:**
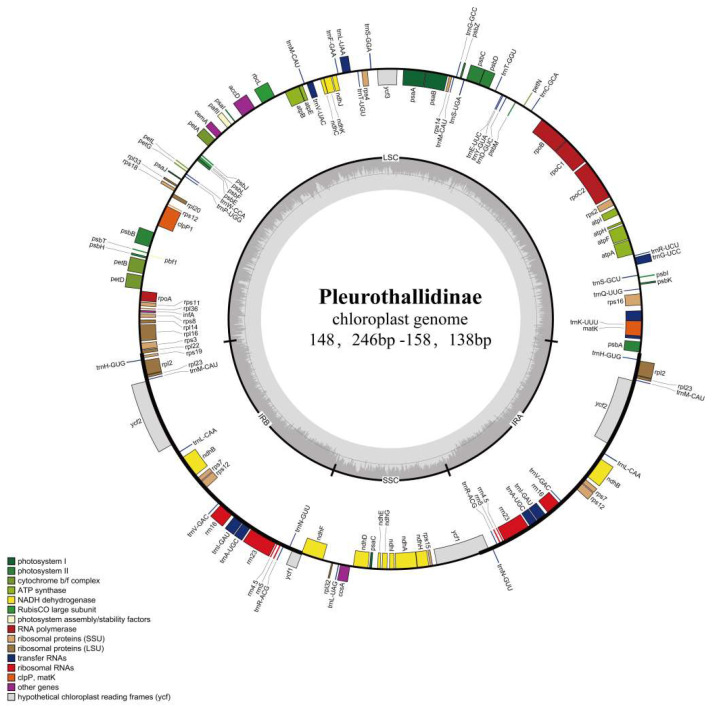
Circular map of the *Dracula erythrochaete* chloroplast genome. Species name, genome length are represented at the center of the plot. The inner circle shows the four regions: the small single-copy (SSC), large single-copy (LSC), and inverted repeat (IRA and IRB) regions. Genes on the outside are transcribed clockwise; those on the inside are transcribed counterclockwise. Different colors are used to distinguish between genes belonging to specific functional categories, and their legend is shown in the lower left corner of the bottom panel. The gray inner circle represents AT content; darker gray represents GC content.

All 25 Pleurothallidinae plastomes exhibited the typical angiosperm quadripartite structure (Figure 1; Table 1). Total length ranged from 148,246 bp (*Acianthera recurva*) to 158,138 bp (*Restrepia trichoglossa*). The LSC region ranged from 83,902 bp (*Anathallis obovata*) to 86,291 bp (*R. trichoglossa*), while the SSC region showed greater variation from 10,573 bp (*Ac.n recurva*) to 21,966 bp (*L. tachirensis*).

Gene content was generally conserved, with 128–134 genes identified including 81–88 protein-coding genes, 37–39 tRNAs, and 8 rRNAs (Table 2). These genes can be categorized into photosynthesis-related genes, self-replication genes, other functional genes, and genes of unknown function. GC content was stable across species (36.7–37.1%), with IR regions more GC-rich (43.1–43.2%) than LSC (34.3–34.8%) or SSC (29.6–30.1%) regions. *Ac. recurva* possessed the smallest plastome with apparent gene losses compared to other species [33,34]. This reduction may be associated with its particular ecological niche, though additional sampling would be needed to determine whether this pattern is species-specific.

### 3.2. IR Boundary Variation

**Figure 2:**
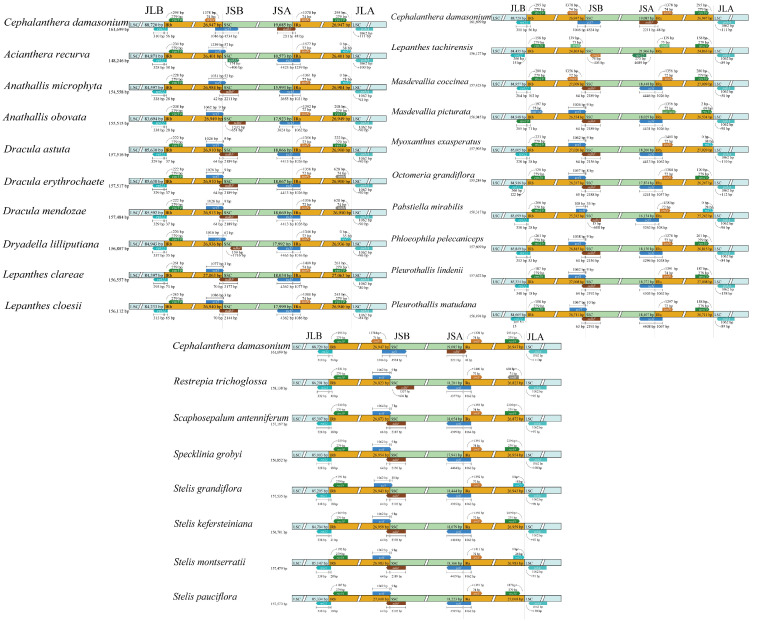
Comparison of IR/SC junction positions among 25 Pleurothallidinae chloroplast genomes, revealing structural variations in the quadripartite chloroplast genome architecture. SSC, the small single-copy region; LSC, large single-copy region; IRA and IRB, inverted repeat regions. JLB, LSC/IRb junction; JSB, IRb/SSC junction; JSA, SSC/IRa junction; JLA, IRa/LSC junction. Numbers indicate distance (bp) from the junction. The numbers inside the boxes represent the lengths of the LSC, SSC, and IRA/B regions. The number alongside the gene name indicates the size (bp) of the gene. The number beside the gene boxes indicates the distance (bp) between the end of the gene and the border sites. Arrows indicate the distance (bp) between the gene and the junction.

Analysis of IR/SC junction positions revealed variation among species (Figure 2). The JLB junction was relatively conserved, with *rpl22* and *rps19* genes consistently present in most species. However, *Masdevallia picturata* showed a distinctive arrangement with *trnH*-*GUG* and *rps19* at this junction, representing a potential species-specific marker.

The JSB and JSA junctions exhibited greater variation. At the JSB junction, *Ac*. *recurva* contained *ycf1* and *rpl32*, *M*. *coccinea* contained *trnN* and *ndhF*, while most other species possessed *ycf1* and *ndhF*. At the JLA junction, species could be grouped into three categories: those with *rpl22* and *psbA*, those with *rps19* and *psbA*, and those with *trnH* and *psbA*.

### 3.3. Repeat Sequence Analysis

**Figure 3:**
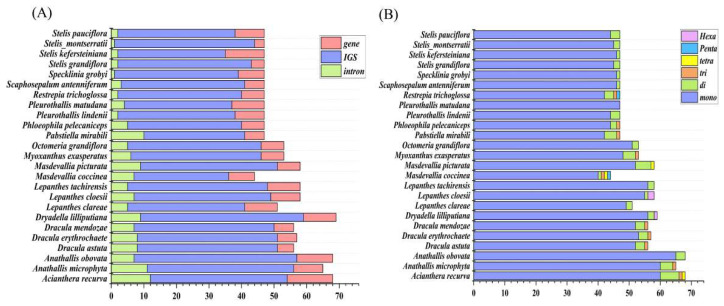
Distribution and types of simple sequence repeats (SSRs) in 25 Pleurothallidinae chloroplast genomes, revealing their distribution patterns, type composition, and abundance. (**A**) Number of SSRs in different genomic regions. (**B**) Number of different SSR types.

Six SSR types were identified across the 25 plastomes (Figure 3). Mononucleotide repeats (predominantly A/T) were most abundant, consistent with patterns in other vascular plants [35,36]. SSRs were mainly distributed in intergenic spacers (29–50 SSRs), followed by coding regions (1–12 SSRs) and introns (3–14 SSRs). Tandem repeat analysis identified 20–60 repeats per genome. Two tandem repeats were located adjacent to functional genes (*rbcL*, *psaI*, *rpl14*, *rps3*) at distances of 133–851 bp (Table 3).

### 3.4. Codon Usage Patterns

**Figure 4:**
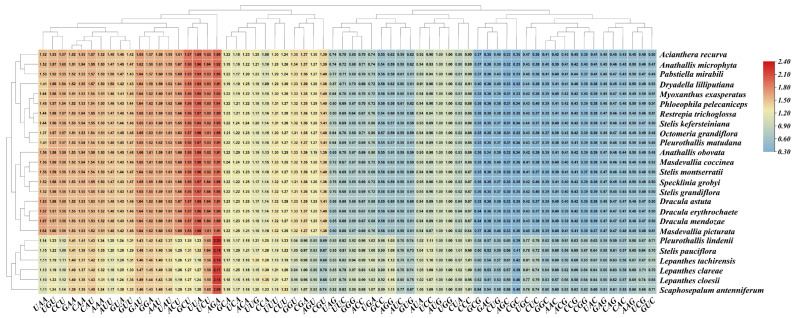
Heatmap of relative synonymous codon usage (RSCU) values for 25 Pleurothallidinae species. Darker green indicates higher RSCU values; darker yellow indicates lower values.

RSCU analysis revealed consistent patterns across species (Figure 4) [37]. Thirty codons exhibited RSCU > 1.0, with codons ending in A or U generally showing higher values than those ending in G or C, reflecting the typical A/T preference of angiosperm plastomes.

**Figure 5:**
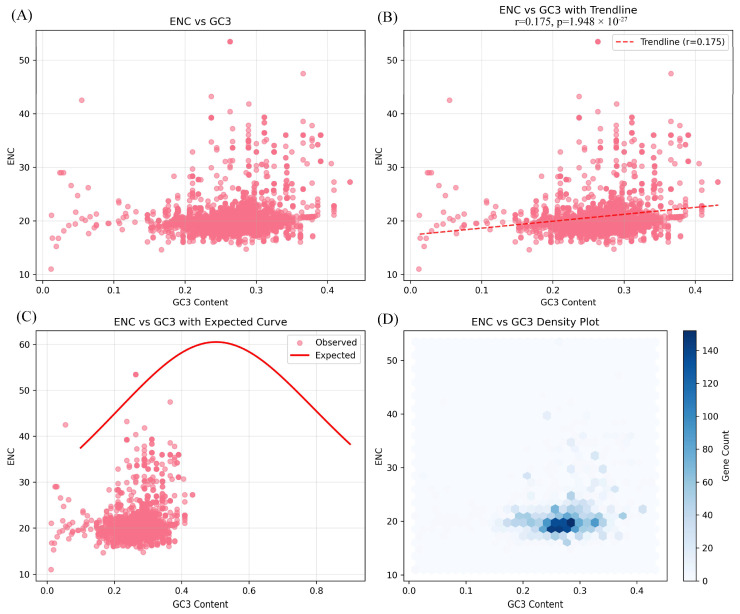
Effective number of codons–GC content at the third codon position (ENC-GC3) plot for protein-coding genes in Pleurothallidinae chloroplast genomes. (**A**) ENC vs. GC3: Distribution of the ENC against the GC3 for all protein-coding genes. (**B**) ENC vs. GC3 with Trendline: The Pearson correlation coefficient (r) and its statistical significance (*p*) are indicated. (**C**) ENC vs. GC3 with Expected Curve: The majority of genes fall below the curve, suggesting a potential influence of natural selection or other factors on codon usage bias. (**D**) ENC vs. GC3 Density Plot. The solid curve represents expected ENC values under mutation pressure alone. Points below the curve indicate selection on codon usage.

Interestingly, we detected differences in codon preferences for CGU, AGU, GGU, CGA, GCU, and UUA among species from different geographic regions. *Pleurothallis* and *Stelis* species from different habitats showed some divergent patterns. These observations suggest that geographic or environmental factors may contribute to codon usage variation. ENC-GC3 analysis showed that most genes fell below the expected neutral curve (Figure 5), which is indicative of a potential role for natural selection in shaping codon usage bias in Pleurothallidinae, beyond the effects of mutational bias alone. However, it should be noted that factors such as mutation bias, GC content, and tRNA abundance could also contribute to the observed patterns, and their relative influences warrant further investigation. This finding is consistent with studies in other orchid lineages showing selection-driven codon bias [38].

### 3.5. Selection Pressure Analysis

**Figure 6:**
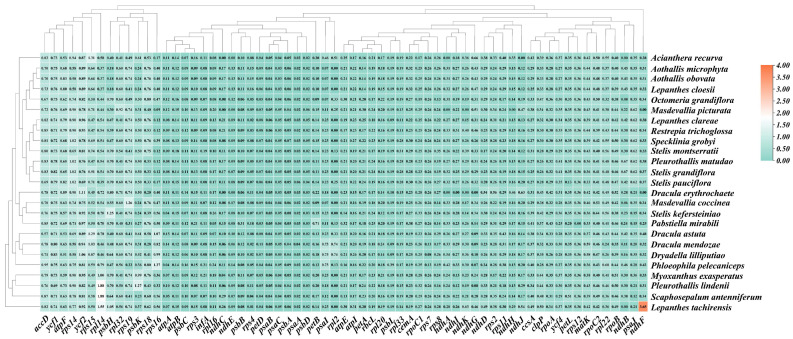
Heatmap of the ratio of non-synonymous to synonymous substitution (Ka/Ks) ratios for protein-coding genes in Pleurothallidinae species. Ka/Ks values were calculated using *C. damasonium* as reference. Darker red indicates higher Ka/Ks; darker green indicates lower values.

Ka/Ks analysis revealed that most protein-coding genes across all species exhibited values <1.0, indicating purifying selection (Figure 6). This pattern reflects functional constraints on plastome-encoded genes essential for photosynthesis and gene expression.

However, we identified several genes with Ka/Ks > 1 in specific lineages. Most notably, *ndhF* in *Lepanthes tachirensis* exhibited Ka/Ks = 3.65, markedly higher than congeners *L*. *cloesii* and *L*. *clareae*. The *ndhF* gene encodes a subunit of the NADH dehydrogenase complex involved in cyclic electron transport. Given that *L*. *tachirensis* is an epiphytic orchid, its life-history strategy inherently imposes stringent demands on photosynthetic regulation. The broad distribution across heterogeneous environments in the Andes further suggests that elevated Ka/Ks ratios in key photosynthetic genes *ndhF*—may signal adaptive evolution in response to heterogeneous combinations of light intensity, drought frequency, and thermal stress across its range. However, Ka/Ks > 1 can also arise from relaxed purifying selection. Elevated Ka/Ks in *ndh* genes have been associated with environmental adaptation in other plant lineages [39].

Additional genes showing Ka/Ks > 1 included: *rpl14* in *L*. *tachirensis*, *Sc. antenniferum*, and *Pleurothallis lindenii*; *rps14* in *Sp. grobyi*, *St. grandiflora*, *Stelis pauciflora*, *Pl*. *matudana*; *rps15* in *Ac*. *recurva*, *Dra. mendozae*, and *Dracula astuta*; *rps16* in *Ph. pelecaniceps* and *Dracula astuta*; *rps19* in *M. coccinea*; *psbH* in *Stelis kefersteiniana*; *psbK* in *Pleurothallis lindenii*; and *ycf2* in *Dracula erythrochaete* and *Dry. lilliputiana*. These lineage-specific patterns suggest that selection pressures on plastome genes may vary among Pleurothallidinae species.

### 3.6. Genome Comparison and Sequence Divergence

**Figure 7:**
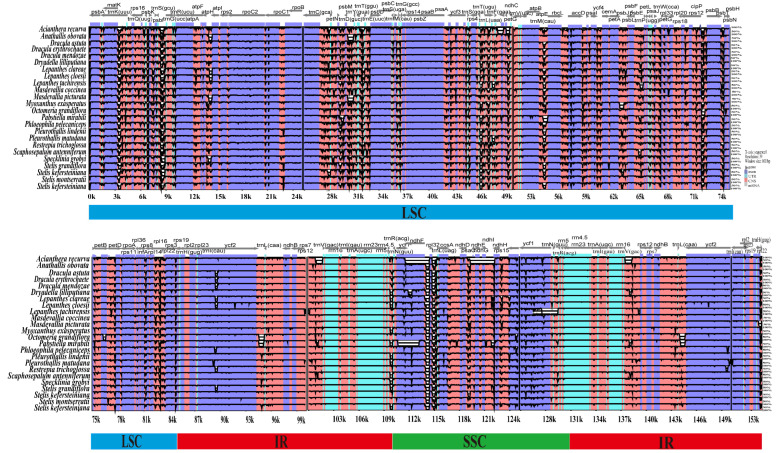
Whole-genome alignment of 24 Pleurothallidinae chloroplast genomes using mVISTA, with *An. microphyta* as reference, identifying divergent and highly conserved regions. Dark blue, exons; light blue, UTR; pink, conserved non-coding sequences. Y-axis indicates percent identity (50–100%).

Whole-genome alignment revealed overall high sequence similarity among the 25 plastomes (Figure 7). Sequence variation was concentrated in LSC and SSC regions, with IR regions more conserved. Non-coding regions showed higher divergence than coding regions. Nine divergent intergenic regions were identified by mVISTA: *matK*-*trnR*-*UCU*, *atpF*-*atpH*, *rpoB*-*psbD*, *ycf3*-*trnV*-*UAC*, *ndhK*-*atpE*, *atpB*-*rbcL*, *accD*-*psaI*, *psbJ*-*psbB*, and *ndhF*-*ccsA*. Notable variation was also detected in *ycf1* [40].

**Figure 8:**
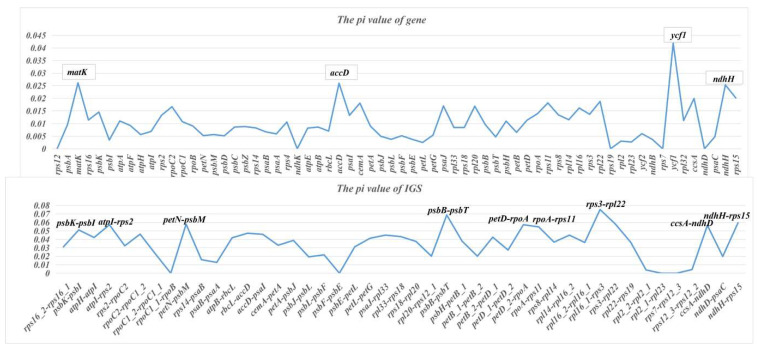
Nucleotide diversity (π) across 25 Pleurothallidinae chloroplast genomes, evaluating hypervariable regions. Sliding window: 600 bp window, 50 bp step. Dashed lines indicate thresholds (π > 0.025 for coding regions; π > 0.05 for intergenic spacers).

Sliding window analysis (window length = 600 bp; step size = 50 bp) identified hypervariable regions suitable for marker development (Figure 8). For coding regions, *matK*, *accD*, *ycf1*, and *ndhH* showed π > 0.025 (more than twice the mean: 0.01). For intergenic spacers, eight regions showed π > 0.05 (exceeding 1.4 times the mean: 0.035): *psbK*-*psbI*, *atpI*-*rps2*, *petN*-*psbM*, *psbB*-*psbT*, *petD*-*rpoA*, *rpoA*-*rps11*, *rps3*-*rpl22*, and *ccsA*-*ndhD*. Therefore, the regions are robust candidate markers identified under the current analytical framework. The identification of hypervariable genomic regions is contingent upon analytical parameters. Excessively large window sizes may dilute localized selective signatures, whereas overly small windows introduce stochastic noise due to limited site sampling. A large step length can overlook narrow peaks of diversity, while a small step increases redundancy without enhancing biological resolution. Moreover, restricted taxon sampling may yield biased diversity estimates due to increased susceptibility to sampling error. In future research, the practical utility in species differentiation or population studies should be further validated by expanding the taxonomic sampling scope and testing under different parameter settings where relevant. Nonetheless, these regions provide candidate markers for species identification and population genetic studies in this taxonomically challenging subtribe [41].

### 3.7. Phylogenetic Relationships

**Figure 9:**
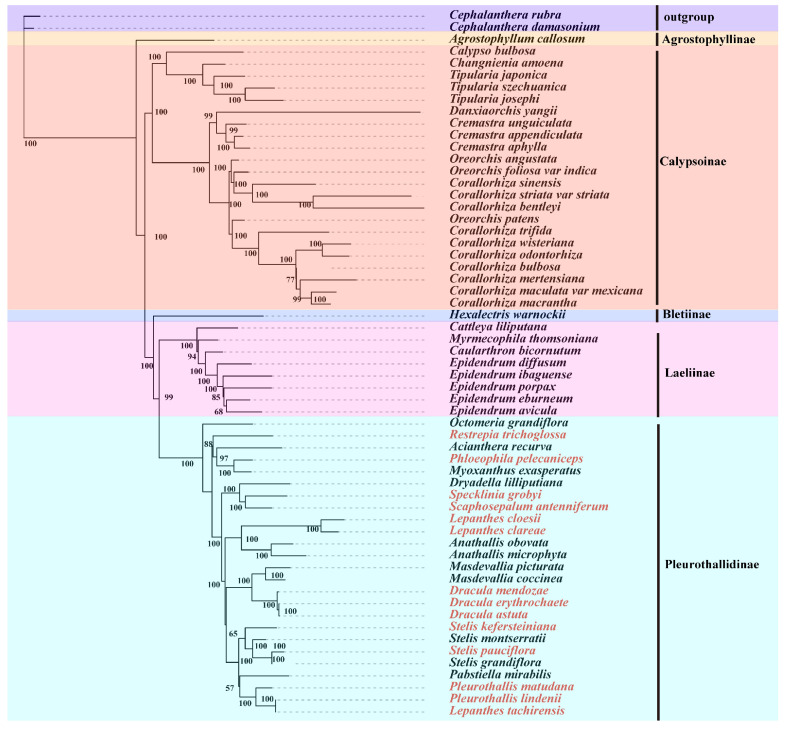
Maximum likelihood phylogenetic tree of 57 Epidendreae species based on chloroplast genomes. *C. damasonium* and *C*. *rubra* were used as outgroups. Bootstrap values (%) are shown at nodes. Red text indicates newly assembled genomes.

Maximum likelihood analysis of 57 Epidendreae species yielded a well-resolved phylogeny with strong bootstrap support (>95% at most nodes; Figure 9). All five subtribes were recovered as monophyletic. Within Pleurothallidinae, 25 species were resolved into six lineages. Consistent with previous studies [2,3], *Lepanthes* and *Pleurothallis* were not monophyletic. *L*. *tachirensis*—traditionally placed in *Lepanthes* subgenus *Lepanthes* subsection Breves [42]—was embedded within a clade containing Pleurothallis species. The phylogenetic tree based on the inverted repeat (IR) regions was consistent with this result (Figure S1). These results further support the need for taxonomic revision of these genera based on molecular evidence.

## 4. Discussion

### 4.1. Plastome Features and Structural Variation

The 25 Pleurothallidinae plastomes analyzed here showed the conserved quadripartite structure typical of angiosperms. Plastome sizes (148,246–158,138 bp) and gene numbers (128–134) are within the range reported for other orchids [11,43]. The strong A/T preference in SSRs is consistent with patterns across vascular plants [34]. IR boundary variation, particularly at JSB and JSA junctions involving *ycf1* and *ndhF*, is common in angiosperms and may provide phylogenetically informative characters [44]. The unique arrangement in *Masdevallia picturata* at the JLB junction could serve as a species-specific marker pending confirmation with additional sampling.

### 4.2. Lineage-Specific Positive Selection and Potential Environmental Adaptation

While most protein-coding genes showed Ka/Ks < 1 indicating purifying selection, we detected signatures of positive selection in specific lineages [45,46,47]. The most striking finding was the elevated Ka/Ks (3.65) for *ndhF* in *L*. *tachirensis*, which contrasts sharply with values in congeners from restricted distribution. The *ndhF* gene product participates in the NDH complex mediating cyclic electron flow around photosystem I, which is 7important for photoprotection and adaptation to fluctuating light conditions [39]. The Andean mountain range, extending from northwestern Venezuela to Peru, encompasses highly heterogeneous habitats with pronounced latitudinal and altitudinal gradients. This environmental diversity results in variable light conditions, UV radiation, and temperature fluctuations, which may exert unique selective pressures on the photosynthetic machinery of resident species [48,49].

This finding is consistent with studies reporting elevated evolutionary rates in *ndh* genes in plant lineages experiencing environmental stress [39]. Although the geographic and ecological context of *L. tachirensis* is consistent with the hypothesis that its elevated Ka/Ks may reflect environmental adaptation, positing a direct adaptive link based on a single species comparison is speculative. Functional validation is therefore required to confirm the adaptive significance of these genetic changes. Future studies with expanded sampling across altitudinal gradients could test whether similar patterns occur in other high-altitude Pleurothallidinae. Therefore, while this anomalous *ndhF* value is noteworthy and merits further investigation, we do not consider habitat heterogeneity conclusive evidence for positive selection.

### 4.3. Codon Usage Bias: Evidence for Selection

ENC-plot analysis indicated that codon usage in Pleurothallidinae deviates from a model governed solely by mutation pressure, indicating a probable contribution of natural selection (e.g., translational efficiency) alongside other factors such as genomic nucleotide composition and mutational spectra [38,50]. This finding has implications for understanding plastome evolution in this subtribe. Selection on codon usage can reflect optimization of translational efficiency, particularly for highly expressed genes.

The observation that species from different geographic regions showed variation in codon preferences for specific codons (CGU, AGU, GGU, CGA, GCU, UUA) is intriguing. While these patterns suggest a potential influence of geographic or environmental factors on codon usage variation, this interpretation should be treated with caution [51]. Equally plausible alternatives for these differences include stochastic evolutionary processes specific to certain lineages, spatially heterogeneous mutation pressures, or the influence of other factors that our phylogenetic framework lacks the power to resolve. If confirmed with broader sampling, this would indicate that plastome evolution in Pleurothallidinae responds to local environmental conditions, potentially through selection for translational efficiency under different temperature or light regimes [52,53,54].

### 4.4. Molecular Markers and Taxonomic Implications

We identified eight hypervariable intergenic regions with π > 0.05 that could serve as molecular markers for species identification and population genetics in Pleurothallidinae. Given that traditional morphology-based identification can be challenging in this subtribe, molecular markers are particularly valuable [55,56,57]. The regions identified here (*psbK*-*psbI*, *atpI*-*rps2*, *petN*-*psbM*, *psbB*-*psbT*, *petD*-*rpoA*, *rpoA*-*rps11*, *rps3*-*rpl22*, *ccsA*-*ndhD*) complement previously reported variable regions and provide additional options for marker development.

Our phylogenetic results confirm the non-monophyly of *Lepanthes* and *Pleurothallis*, consistent with earlier molecular studies [2,3]. The placement of *L*. *tachirensis* within a Pleurothallis-dominated clade, highlighting a critical limitation of traditional taxonomic frameworks: the floral characters long relied upon to delimit these genera, such as the degree of sepal fusion, labellum morphology, and structural differentiation of floral segments, do not consistently track the evolutionary relationships inferred from molecular data. This discrepancy arises primarily from the high level of homoplasy in floral traits within Pleurothallidinae [2]. These findings further validate the necessity of ongoing taxonomic revisions that integrate molecular phylogenetics with morphological reevaluation [2,19], as they underscore the inadequacy of relying solely on floral characters for generic delimitation in this evolutionarily dynamic subtribe.

The relatively long branch length of *Lepanthes* in the chloroplast genome coding region-based phylogenetic tree is closely linked to its molecular evolutionary patterns and taxonomic delimitation, and also provides a reference for the screening of molecular markers within this group. Previous studies have shown that the early diversification of the *Lepanthes* lineage (>1500 species)—dated to around 8 Ma—is associated with the colonization of novel Andean habitats, rapid mountain uplift, and the emergence of specialized pollination systems (e.g., pseudocopulation and food mimicry, reported in closely related taxa) [3,28,58]. The accelerated evolutionary rate in *Lepanthes* likely drove extensive accumulation of synonymous mutations, thereby amplifying the genetic distance between the *Lepanthes* clade and related groups and resulting in the observed long-branch phenomenon in phylogenetic reconstructions. Future studies should further verify whether synonymous mutation accumulation is the main cause of branch elongation by performing phylogenetic reconstructions using amino acid sequences from chloroplast coding regions, which can also alleviate the confounding effects of non-synonymous mutations on branch length and taxonomic inference. Meanwhile, integrating nuclear gene sequences and other molecular markers will help clarify the taxonomic boundaries and evolutionary history of *Lepanthes*. These efforts will provide more reliable molecular evidence for taxonomic revision in this group.

### 4.5. Limitations and Future Directions

Several limitations should be noted. Our sampling represents a subset of Pleurothallidinae diversity, and patterns observed here may not apply to all lineages. The elevated Ka/Ks values should be interpreted cautiously pending functional validation. The correlation between codon usage and geography requires confirmation with broader sampling. Future studies should expand taxonomic and geographic coverage, validate putative adaptive signatures with functional assays, and integrate nuclear genomic data for comprehensive evolutionary analysis.

## 5. Conclusions

We analyzed 25 plastomes representing 15 genera of Pleurothallidinae, including 14 newly assembled genomes. All plastomes showed a conserved quadripartite structure with predominantly purifying selection on protein-coding genes. We identified lineage-specific signatures of positive selection, most notably in *ndhF* of the high-altitude species *L*. *tachirensis* (Ka/Ks = 3.65), suggesting potential environmental adaptation. Codon usage in Pleurothallidinae shows a marked departure from the mutation-dominated neutral model, demonstrating that natural selection plays a potential role in driving codon evolution, with distinct patterns diverging across species from different geographic regions. We identified eight hypervariable intergenic regions suitable as molecular markers, and confirmed that *Lepanthes* and *Pleurothallis* are non-monophyletic. These findings expand genomic resources for Pleurothallidinae, reveal patterns of lineage-specific plastome evolution, and provide molecular tools for future taxonomic and evolutionary studies.

## Figures and Tables

**Table 1:** Summary of complete chloroplast genomes of 25 Pleurothallidinae species, including region lengths, GC content, and gene composition. LSC, large single-copy region; SSC, small single-copy region; and IR, inverted repeat region.

	Length (bp)				Number of Genes				GC%	Accession
	LSC Size	SSC Size	IR Size	Genome Size	tRNA	rRNA	Protein Coding	Total	Total GC Content/%	
*Ac. recurva*	84,871	10,573	26,401	14,8246	38	8	81	128	37.00%	MW375123
*An. microphyta*	84,597	15,993	26,984	15,4558	38	8	85	132	37.00%	MW375124
*Anathallis obovata*	83,902	19,307	26,749	15,5515	38	8	87	133	37.10%	MH979332
*Dracula mendozae*	85,592	18,069	26,910	15,7484	38	8	86	132	36.90%	OR713737
*Dracula erythrochaete*	85,630	18,067	26,910	15,7517	38	8	86	132	36.90%	OR713738
*Dracula astuta*	85,630	18,066	26,910	15,7516	39	8	88	134	36.90%	PP094516
*Dryadella lilliputiana*	84,943	17,992	26,936	15,6807	38	8	87	134	37.10%	MW375126
*Lepanthes clareae*	84,397	18,034	27,063	15,6557	38	8	87	133	37.10%	PX828354
*Lepanthes cloesii*	84,233	17,999	26,940	15,6112	38	8	84	130	37.10%	PX828355
*L. tachirensis*	84,435	21,966	24,863	15,6127	38	8	84	130	37.10%	PX828356
*Masdevallia coccinea*	84,957	18,448	27,009	15,7423	38	8	86	132	36.80%	NC_026541
*Masdevallia picturata*	84,948	18,029	26,534	15,6045	37	8	88	133	36.90%	KJ566305
*Myoxanthus exasperatus*	85,605	18,260	27,020	15,7905	38	8	87	134	37.10%	MW375127
*Octomeria grandiflora*	84,916	17,874	26,247	15,5284	38	8	87	133	36.90%	MW375128
*Pabstiella mirabilis*	83,699	16,134	25,242	15,0317	38	8	86	133	37.10%	MW375130
*Phloeophila pelecaniceps*	85,849	18,130	26,815	15,7609	38	8	87	133	37.10%	OR909688
*Pleurothallis matudana*	84,665	18,407	26,711	15,6494	39	8	87	134	37.00%	OR909687
*Pleurothallis lindenii*	85,334	18,272	27,008	15,6127	38	8	85	131	36.90%	PX828359
*R. trichoglossa*	86,291	18,201	26,823	15,8138	38	8	86	132	36.70%	OR713739
*Scaphosepalum antenniferum*	85,397	18,054	26,873	15,7197	38	8	86	132	37.00%	PX828357
*Specklinia grobyi*	85,003	17,941	26,954	15,6852	38	8	87	133	37.00%	OR713740
*Stelis grandiflora*	85,205	18,444	26,943	15,7535	38	8	87	134	36.90%	MW375129
*Stelis kefersteiniana*	84,704	18,079	26,959	15,6701	38	8	87	133	36.90%	OR909686
*Stelis montserratii*	85,174	18,366	26,983	15,7479	38	8	86	133	36.90%	MW375125
*Stelis pauciflora*	85,334	18,223	27,008	15,7573	38	8	87	133	36.90%	PX828358

**Table 2:** Gene composition of chloroplast genomes in Pleurothallidinae species, including gene content, functional categories, and conservation patterns. Genes are classified according to their functions.

Category	Gene Group	Gene Name
*Photosynthesis*	*Subunits of photosystem I*	*psaA*, *psaB*, *psaC*, *psaI*, *psaJ*
	*Subunits of photosystem II*	*psbA*, *psbB*, *psbC*, *psbD*, *psbE*, *psbF*, *psbH*, *psbI*, *psbJ*, *psbK*, *psbL*, *psbM*, *psbT*, *psbZ*
	*Subunits of NADH dehydrogenase*	ndhA * (2), ndhB * (2), *ndhC*, *ndhD*, *ndhE*, *ndhF*, *ndhG*, *ndhH*, *ndhI*, *ndhJ*
	*Subunits of cytochrome b/f complex*	*petA*, petB *, *petD*, petD *, *petG*, *petL*, *petN*
	*Subunits of ATP synthase*	*atpA*, *atpB*, *atpE*, *atpF*, atpF *, *atpH*, *atpI*
	*Large subunit of rubisco*	*rbcL*
	*Subunits of photochlorophyllide reductase*	*-*
*Self-replication*	*Proteins of large ribosomal subunit*	*# rpl14*, rpl16 *, *rpl2(2)*, rpl2 *(2), *rpl20*, *rpl22*, *rpl23(2)*, *rpl32*, *rpl33*, *rpl36*
	*Proteins of small ribosomal subunit*	*rps11*, *rps12*, rps12 ** (2), *rps14*, *rps15*, rps16 *, *rps18*, *rps19(2)*, *rps2*, *rps3*, *rps4*, *rps7(2)*, *rps8*
	*Subunits of RNA polymerase*	*rpoA*, *rpoB*, *rpoC1*, rpoC1 *, *rpoC2*
	*Ribosomal RNAs*	*rrn16(2)*, *rrn23(4)*, *rrn4.5(2)*, *rrn5(2)*
	*Transfer RNAs*	*trnA-UGC(2)*, trnA-UGC * (4), *trnC-GCA*, *trnD-GUC(2)*, *trnE-UUC(2)*, trnE-UUC * (2), *trnF-GAA*, *trnG-GCC(2)*, trnG-UCC *, *trnH-GUG(6)*, trnI-GAU * (2), trnK-UUU *, *trnL-CAA(4)*, trnL-UAA * (2), *trnL-UAG*, *trnM-CAU|trnI-CAU(2)*, *trnM-CAU|trnfM-CAU*, *trnM-CAU(4)*, *trnN-GUU(4)*, *trnP-UGG(2)*, *trnQ-UUG*, *trnR-ACG(4)*, *trnR-UCU*, trnS-CGA *, *trnS-GCU*, *trnS-GGA*, *trnS-UGA(2)*, *trnT-GGU(2)*, *trnT-UGU(2)*, *trnV-GAC(2)*, trnV-UAC *, *trnW-CCA*, *trnY-GUA(2)*
*Other genes*	*Maturase*	*matK(2)*
	*Protease*	clpP **
	*Envelope membrane protein*	*cemA*
	*Acetyl-CoA carboxylase*	*accD*
	*c-type cytochrome synthesis gene*	*ccsA*
	*Translation initiation factor*	*infA*
	*other*	*pbf1*
*Genes of unknown function*	*Conserved hypothetical chloroplast ORF*	*ycf1(2)*, *ycf2(2)*, ycf3 ** (2), *ycf4*

Note: Asterisks indicate genes with introns (* one intron; ** two introns). Numbers in parentheses indicate copy number. The hash symbol (#) indicates pseudogenes.

**Table 3:** Tandem repeats identified adjacent to functional genes in Pleurothallidinae chloroplast genomes.

TR Name	TR Position (Chr: Start–End)	Period Size	Copy Number Variation Range	Neighboring Gene	Distance (bp)	Gene Function
TR 1	56,521–56,573	21	2.5	*rbcL*, *psaI*	808–851	*Photosynthesis*
TR 2	82,784–82,820	13	2.7	*rpl14*, *rps3*	133–183	*Self-replication*

## Data Availability

Newly assembled chloroplast genome sequences have been deposited in GenBank under accession numbers OR713737–OR713740, OR909686–OR909688, PP094516 and PX828354–PX828359.

## Supplementary Materials

The following supporting information can be downloaded at: https://www.mdpi.com/article/10.3390/genes17020199/s1, Figure S1: Maximum likelihood phylogenetic tree of 57 Epidendreae species based on inverted repeat (IR) regions; Table S1: ENC-plot values for all protein-coding genes; Table S2: Nucleotide diversity (π) values for coding genes and intergenic spacers among 25 Pleurothallidinae species; Table S3: GenBank accession numbers for species of Agrostophyllinae, Bletiinae, Laeliinae, and Calypsoinae used in phylogenetic analysis.
