# Supplementary figures and images for: Chloroplast Genome Evolution in Pleurothallidinae (Orchidaceae): Lineage-Specific Selection, Codon Usage Patterns, and Phylogenetic Implications
Source: Genes (Basel). 2026 Feb 7;17(2):199. doi: 10.3390/genes17020199 (PMC12941346; doi:10.3390/genes17020199)

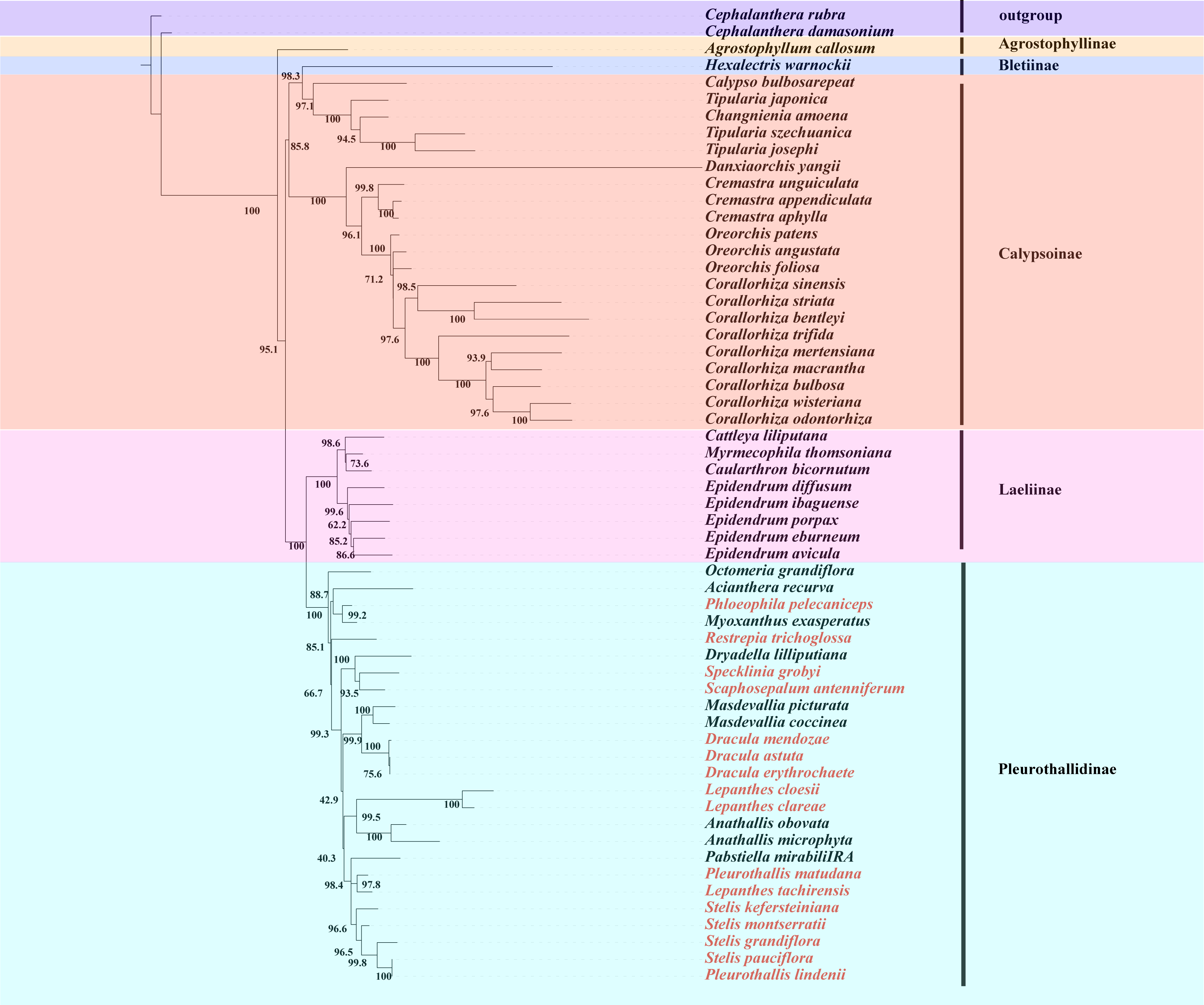

Supplement: Supplementary file 1 [file genes-17-00199-s001.zip › Figure S1.png]
